# A Formative Evaluation of a Diabetes Prevention Program Using the RE-AIM Framework in a Learning Health Care System, Utah, 2013–2015

**DOI:** 10.5888/pcd14.160556

**Published:** 2017-07-20

**Authors:** Kimberly D. Brunisholz, Jaewhan Kim, Lucy A. Savitz, Mia Hashibe, Lisa H. Gren, Sharon Hamilton, Kelly Huynh, Elizabeth A. Joy

**Affiliations:** 1 Intermountain Healthcare, Salt Lake City, Utah; 2 University of Utah, Salt Lake City, Utah

## Abstract

**Introduction:**

Evaluation of interventions can help to close the gap between research and practice but seldom takes place during implementation. Using the RE-AIM framework, we conducted a formative evaluation of the first year of the Intermountain Healthcare Diabetes Prevention Program (DPP).

**Methods:**

Adult patients who met the criteria for prediabetes (HbA1c of 5.70%–6.49% or fasting plasma glucose of 100–125 mg/dL) were attributed to a primary care provider from August 1, 2013, through July 31, 2014. Physicians invited eligible patients to participate in the program during an office visit. We evaluated 1) reach, with data on patient eligibility, participation, and representativeness; 2) effectiveness, with data on attaining a 5% weight loss; 3) adoption, with data on providers and clinics that referred patients to the program; and 4) implementation, with data on patient encounters. We did not measure maintenance.

**Results:**

Of the 6,862 prediabetes patients who had an in-person office visit with their provider, 8.4% of eligible patients enrolled. Likelihood of participation was higher among patients who were female, aged 70 years or older, or overweight; had depression and higher weight at study enrollment; or were prescribed metformin. DPP participants were more likely than nonparticipants to achieve a 5% weight loss (odds ratio, 1.70; 95% confidence interval, 1.29–2.25; *P* < .001). Providers from 7 of 8 regions referred patients to the DPP; 174 providers at 53 clinics enrolled patients. The mean number of DPP counseling encounters per patient was 2.3 (range, 1–16).

**Conclusion:**

The RE-AIM framework was useful for estimating the formative impact (ie, reach, effectiveness, adoption, and implementation fidelity) of a DPP-based lifestyle intervention deployed in a learning health care system.

## Introduction

An estimated 86 million adults, or more than one-third of Americans, have prediabetes and are at high risk for developing type 2 diabetes; however, only 1 in 10 adults in the Unites States has been told by a health care provider that he or she has the condition (1). Diabetes results from a combination of genetic predisposition and behavioral and environmental risk factors. However, there is strong evidence that such modifiable risk factors as poor nutrition, obesity, and physical inactivity are the main environmental determinants of the disease (2).

Several clinical trials have shown intensive lifestyle interventions to be efficacious in attaining and maintaining weight loss, which is a key to preventing progression to diabetes for those at risk for disease (3–8). The Diabetes Prevention Program Outcomes Study demonstrated that lifestyle or metformin interventions can delay onset of diabetes for 10 years, suggesting that clinical improvements are not just transient effects (9). Although effective interventions may require a large investment, simulation models show that a national prevention program would break even in 14 years and prevent or delay 885,000 cases of diabetes in the United States within 25 years, producing a cost savings of $5.7 billion (10). A study evaluating a weight-management program from the Department of Veterans Affairs showed that the slope of weight among participants improved significantly from preintervention to postintervention (11). Further study indicated low rates of participation among eligible veterans and low levels of weight loss when the program used provider-based referrals (12). Other Diabetes Prevention Program (DPP) translational efforts demonstrated high attendance rates and low attrition rates when participants were invited by a trusted health professional (13), effective weight loss when the program was delivered by trained diabetes educators (14,15), and sustainability when the program was implemented in a community setting (7, 16–18).

Most studies using the RE-AIM (reach, effectiveness, adoption, implementation, maintenance) framework to evaluate diabetes interventions focused on internal validity, reach, and effectiveness, not on adoption or implementation (19,20). Little evidence exists on diabetes interventions performed in real-world settings.

In 2013, Intermountain Healthcare, a Utah-based nonprofit health care system (21), created a DPP for patients at risk for type 2 diabetes. The objective of this study was to describe the reach, effectiveness, adoption, and implementation of the Intermountain Healthcare DPP.

## Methods

We conducted a formative evaluation of the Intermountain Healthcare DPP in Salt Lake City, Utah, during its first year of implementation (August 1, 2013, through July 31, 2014, hereinafter, “study period”) using the RE-AIM framework (22,23). We did not measure maintenance in this study because of the longer time required to do so. The institutional review board at Intermountain Healthcare approved this study.

### Intermountain Healthcare’s DPP

In early 2013, Intermountain Healthcare began to plan a DPP for its primary care clinics (K.D.B., unpublished data, 2015). A modified form of the national DPP, the Intermountain Healthcare DPP comprises 3 ways to participate: 1) an introductory 2-hour group class (Prediabetes 101), 2) individual nutrition counseling sessions (medical nutrition therapy, or MNT), and 3) a hospital-based behavioral program, offered in 12 classes during 6 months (Weigh to Health, or W2H). After discussing treatment options with their provider and care team, patients could select an option that would work best for their needs and preferences or elect not to participate. Patients could choose to participate in any or all options (24).

From August to December 2013, we conducted feasibility studies in 5 clinics to test the deployment of Prediabetes 101. MNT and W2H had already been operating and were added as options to the DPP. On the basis of the results of the feasibility studies, the DPP workgroup 1) changed the process for recruiting participants from calling them on the telephone to extending an invitation during an office visit, 2) defined distinct roles and responsibilities for the clinical and DPP teams, 3) standardized the referral process and DPP documentation for the 3 DPP options in the electronic medical record, and 4) conducted regional trainings and clinical in-services for providers and clinical staff. Prediabetes 101 was used as a patient engagement tool and was free of charge to all patients in the system. MNT and W2H continued to require insurance coverage to be approved on a patient-by-patient basis. The DPP was fully deployed in January 2014.

Patients who had a diagnosis of prediabetes (hemoglobin A1c [HbA1c] of 5.70%–6.49% or fasting plasma glucose of 100–125 mg/dL) recorded in the Intermountain Healthcare enterprise data warehouse during the study period were considered eligible to participate in the DPP. Beginning August 1, 2013, providers were encouraged to invite eligible patients to participate during their next office visit. Patients who attended a Prediabetes 101 class, MNT, or W2H during the study period were assigned to the intervention (DPP) group. Patients with prediabetes who were attributed to the same group of primary care physicians as the intervention group and had an opportunity to be invited by their primary care physician (but were not) were assigned to the control (no DPP) group.

### RE-AIM evaluation framework

The RE-AIM framework was developed to enhance the impact of health promotion interventions by evaluating the dimensions considered most relevant to real-world implementation (22,23,25). “Reach” refers to the percentage and characteristics of people receiving the intervention; “effectiveness” refers to the impact of the intervention, including anticipated and unanticipated outcomes; “adoption” refers to the percentage and representativeness of settings that adopt the intervention; “implementation” refers to the consistency and cost of delivering the intervention; and “maintenance” refers to long-term sustainability in the setting and among individuals.

### Data set and sources

**Analytic data set.** Of patients who had a diagnosis of prediabetes, we excluded from analysis patients who did not have an office visit with a provider during the study period. Of patients who had an office visit and thus an opportunity to be invited by a physician to participate, we excluded the following patients from analysis: patients who received a diagnosis of diabetes before enrollment or within 2 months after enrollment, whose diagnosis of prediabetes was incorrect, who declined to participate, who had a medical condition not related to weight loss or diabetes prevention, who had already begun weight-loss education, who had weight-loss surgery, who were aged younger than 18, or who died during the study period.

**Data collection.** We collected data on participants from Intermountain Healthcare’s enterprise data warehouse on demographic characteristics (age, sex, race/ethnicity, and insurance status) and clinical characteristics (duration of prediabetes diagnosis; diagnosis of 5 chronic diseases before enrollment in the DPP [atrial fibrillation, congestive heart failure, coronary artery disease, depression, and high blood pressure]; whether taking antihypertensive medications, atypical neuroleptics, metformin, or statins; and weight and body mass index [BMI] class at study enrollment). Chronic conditions were based on diagnosis codes (Appendix) and encounter data and were approved by an internal expert committee of providers. Duration of prediabetes was estimated from the first documentation of laboratory values in the enterprise data warehouse. Data on use of the 4 medications were collected at study enrollment.

Intermountain Healthcare is organized into 8 regions, based on travel and referral patterns. Each region is anchored by hospitals, specialty and primary care clinics, and home health, and consists of both rural and urban communities. Patients were attributed annually to their provider and clinic, which defined the number of participating and nonparticipating patients at each clinic.

### Assessment and statistical analyses of reach

Reach was defined as the number of participants who enrolled in the DPP (numerator) divided by the number eligible to participate in the DPP (denominator). Because the DPP may have been implemented differently in each region, we stratified data by region. Representativeness was based on comparisons of participants to nonparticipants for demographic characteristics, clinical characteristics, and regional operational characteristics. To determine the independent associations between DPP participation and patient characteristics, we used multivariable logistic regression modeling. We included the following potential confounders in the logit model to predict the likelihood of treatment (ie, DPP participation): age, sex, race/ethnicity, insurance, duration of prediabetes, weight at study enrollment, BMI class, medication use, and prevalence of atrial fibrillation, congestive heart failure, coronary artery disease, depression, and high blood pressure. Subsequently, we conducted posthoc analyses to adjust for possible variation in program implementation by clinic using mixed-effects logistic modeling. We also included in the model the following regional operational characteristics: number of patients attributed to the clinic, number of providers, urban or rural location, and level of medical home adoption (26).

### Assessment and statistical analyses of effectiveness

To assess effectiveness, we determined the association of 5% weight loss and the incidence of type 2 diabetes among participants and nonparticipants. Initially, patients were matched on a 1:4 ratio (intervention to control) based on a propensity score method that uses a nearest-neighbor technique (27). This method was operationalized by first including potential confounders in a logit model to predict the propensity for treatment (ie, DPP participation). Characteristics considered as potential confounders were age, sex, race/ethnicity, duration of prediabetes, weight at study enrollment, and prevalence of atrial fibrillation, congestive heart failure, coronary artery disease, depression, and high blood pressure. This weighting method produced estimates for an “average treatment effect on the treated,” answering the question: “Among control patients closely resembling the DPP patients, what outcomes were associated with the intervention?” (28).

To determine which patients achieved a 5% weight loss, we collected data on weight within 12 months before study enrollment and data on follow-up weight within 5 to 7 months after enrollment. We calculated changes in weight to determine a binary outcome (yes or no) of whether a patient achieved a 5% weight loss from study enrollment. We also determined incident diagnosis of type 2 diabetes (yes or no) according to Healthcare Effectiveness Data and Information Set (HEDIS) specifications (29), which require a diagnosis code of diabetes (*International Classification of Diseases, 9th Revision, Clinical Modification,* code 250) and one of the following: 1) 2 outpatient encounters on different dates of service, 2) 1 acute inpatient encounter, 3) 1 emergency department visit, or 4) a prescription for insulin or hypoglycemic/antihyperglycemic medication on an ambulatory basis.

We used conditional logistic regression modeling to obtain summary measures of relative risk for the study groups. We generated odds ratios [ORs] after adjusting for differences at study enrollment, including demographic and clinical characteristics (through the use of a propensity score that was produced in the first model) that are known to affect the ability to achieve 5% weight loss. Similarly, we used this method to determine the incidence of type 2 diabetes in groups. We used difference-in-difference modeling to measure the association and magnitude measurement of weight change from study enrollment to follow-up.

### Assessment of adoption and implementation

To assess adoption, we calculated the number of providers and clinics that referred patients to the program and the range of the number of patients referred per provider. To evaluate implementation, we used a proxy measure of fidelity to the DPP process. We assessed the mean number of encounters per patient by each DPP option and the proportion of patients who had only 1 encounter. An encounter was defined as a visit during the program. We assessed the proportion of patients who enrolled in W2H; we defined completeness as having 12 or more encounters in W2H. For all analyses, we considered a 2-sided *P* value of .05 or less to be significant. We analyzed all data using Stata version 12.0 (StataCorp LLC).

## Results

**Reach.** During the study period, 17,142 people met the criteria for prediabetes; 6,862 were considered eligible for the DPP program because they met the study criteria, had an in-person office visit with their provider, and had the opportunity to be invited to the program (Figure). During the study period, 573 (8.4%) patients participated in the DPP. Of these, 384 (67%) participated in Prediabetes 101, 213 (37%) in MNT, and 54 (9%) in W2H; 63 patients participated in more than 1 DPP option, and all participated in either Prediabetes 101 or MNT. The DPP participation rate was greatest for region 2 (13.2%), region 5 (10.6%), and region 6 (12.6%).

**Figure:**
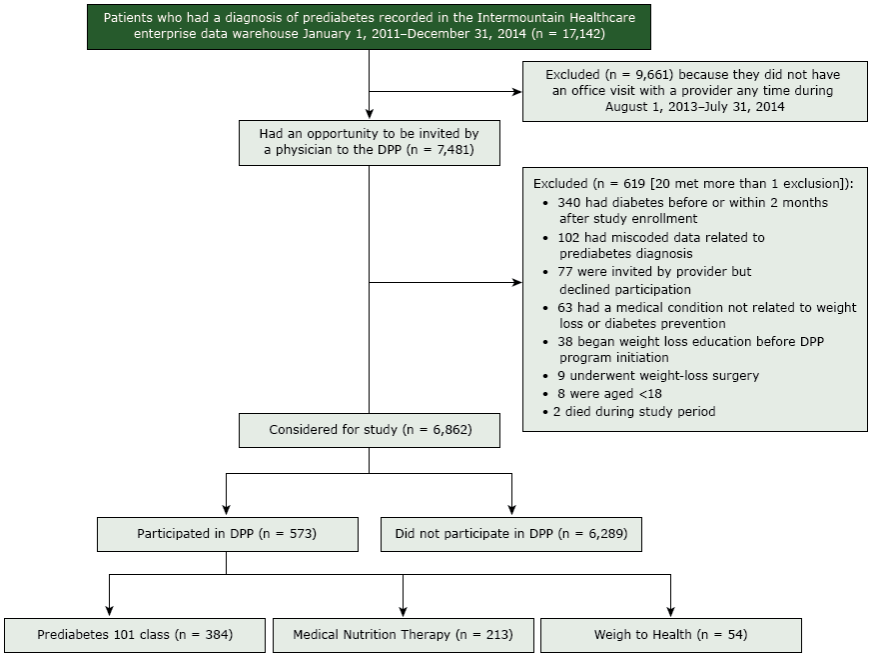
Flow of participants through a diabetes prevention program (DPP) at Utah-based Intermountain Healthcare, 2013–2014. Patients could participate in more than 1 type of class.

After adjustment for demographic and clinical characteristics, the following characteristics were associated with a greater likelihood of participation in the DPP: female sex, age of 70 years or older, overweight, depression, higher weight at study enrollment, and a prescription for metformin (Table 1). Likelihood of participation was lower among patients whose prediabetes was diagnosed 5 to 9 years previously than among patients whose prediabetes was diagnosed less than 5 years previously. Posthoc analyses confirmed that patients who were female, had a higher weight at study enrollment, or had a prescription for metformin or a medication for hypertension were more likely to participate (Table 2).

**Table 1:** Measurement of Reach: Characteristics and Associations of Patients Who Participated in a Diabetes Prevention Program, Intermountain Healthcare, 2013–2014

Variables	No. of Patients Who Met Study Criteria^a^ (n = 6,862)	No. (%)^b^ of DPP Participants (n = 573)	No. (%)^b^ of Nonparticipants (n = 6,289)	Multivariable-Adjusted Associations of Participation^c^, OR (95% CI)
**Demographic Characteristics**				
**Age, y**				
18–29	174	19 (3.3)	155 (2.5)	1 [Reference]
30–39	506	39 (6.8)	467 (7.4)	0.69 (0.40–1.17)
40–49	982	76 (13.3)	906 (14.4)	0.78 (0.44–1.37)
50–59	1,916	165 (28.8)	1,751 (27.9)	0.91 (0.53–1.56)
60–69	2,357	151 (26.4)	2,206 (35.1)	0.70 (0.40–1.21)
≥70	919	123 (21.5)	796 (12.7)	1.75 (1.08–2.83)^d^
**Sex**				
Male	3,070	212 (37.3)	2,858 (45.4)	1 [Reference]
Female	3,787	356 (62.7)	3,431 (54.6)	1.43 (1.09–1.88)^d^
**Race/ethnicity**				
Non-Hispanic white	5,947	506 (88.4)	5,441 (86.5)	1 [Reference]
Hispanic	575	44 (7.6)	531 (8.4)	0.73 (0.39–1.35)
Non-Hispanic black	57	4 (0.7)	53 (0.8)	0.80 (0.27–2.35)
Asian	145	10 (1.8)	135 (2.2)	0.98 (0.70–1.38)
Other	138	9 (1.5)	129 (2.0)	0.72 (0.46–1.14)
**Insurance**				
Commercial	3,967	294 (51.3)	3,673 (58.4)	1 [Reference]
Medicare	2,208	194 (33.9)	2,014 (32.0)	0.92 (0.77–1.09)
Medicaid	272	21 (3.7)	251 (4.0)	0.84 (0.51–1.38)
Uninsured	415	64 (11.2)	351 (5.6)	2.21 (0.85–5.74)
**Clinical Characteristics**				
**Prediabetes duration, y**				
<5	5,389	458 (79.9)	4,931 (78.4)	1 [Reference]
5–9	1,303	100 (17.5)	1,203 (19.1)	0.71 (0.56–0.92)^d^
≥10	170	15 (2.6)	155 (2.5)	0.86 (0.49–1.49)
**Chronic conditions^e^**				
Depression	2,808	272 (47.4)	2,536 (40.3)	1.15 (1.03–1.28)^d^
Coronary artery disease	1,234	114 (19.9)	1,120 (17.8)	1.13 (0.76–1.70)
Congestive heart failure	516	51 (9.0)	465 (7.4)	0.93 (0.67–1.29)
Atrial fibrillation	416	45 (7.9)	371 (5.9)	1.28 (0.91–1.81)
High blood pressure	3,908	336 (58.7)	3,572 (56.8)	1.12 (0.92–1.35)
**Medication class^f^**				
Antihypertensive	3,322	274 (47.8)	3,048 (48.5)	0.81 (0.54–1.21)
Atypical neuroleptic	576	55 (9.6)	521 (8.3)	1.02 (0.81–1.29)
Metformin	1,110	124 (21.6)	986 (15.7)	1.36 (1.01–1.87)^d^
Statin	3497	304 (53.1)	3,193 (50.8)	0.97 (0.86–1.11)
**Weight at study enrollment, no. of patients (mean [SD] kg)**	6,862	573 (98.8 [25.4])	6,289 (97.0 [25.4])	1.01 (1.00–1.01)^d^
**BMI class at study enrollment (kg/m^2^)**				
Underweight (<18.5)	40	2 (0.4)	38 (0.6)	0.81 (0.26–2.49)
Normal (18.5–24.9)	686	38 (7.2)	648 (10.7)	1 [Reference]
Overweight (25.0–29.9)	1,679	130 (24.8)	1,549 (25.5)	1.39 (1.02–1.89)^d^
Obese (≥30.0)	4,193	355 (67.6)	3,838 (63.2)	1.21 (0.68–2.13)

Abbreviations: BMI, body mass index; CI, confidence interval; DPP, diabetes prevention program; HbA1c, hemoglobin A1c; OR, odds ratio; SD, standard deviation.

a After all inclusion and exclusion criteria were applied to 17,142 patients who had a diagnosis of prediabetes (HbA1c of 5.70%–6.49% or fasting plasma glucose of 100–125 mg/dL) recorded in the Intermountain Healthcare enterprise data warehouse. Numbers in each category may not add to 6,862 because data were missing for some items.

b Values are number (percentage) except for weight at study enrollment. Percentages in each category may not add to 100 because of rounding or because patients could have more than 1 chronic condition or take more than 1 type of medication.

c All covariates listed in the table were included in the model.

d *P* < .05.

e The reference group for each chronic condition is the group of patients who did not have the condition.

f The reference group for each medication class is the group of patients who were not taking the medication.

**Table 2:** Mixed-Effects Logistic Regression Modeling to Predict Participation in a Diabetes Prevention Program, Intermountain Healthcare, 2013–2014

Variables	No. of Patients Who Met Study Criteria^a^ (n = 6,862)	No. of DPP Participants (n = 573)	OR (95%CI)	*P* Value
**Demographics**				
**Age, y**				
18–29	174	19	1 [Reference]	
30–39	506	39	0.67 (0.36–1.24)	.21
40–49	982	76	0.79 (0.44–1.40)	.42
50–59	1,916	165	0.85 (0.49–1.49)	.57
60–69	2,357	151	0.67 (0.37–1.21)	.19
≥70	919	123	1.62 (0.85–3.10)	.14
**Sex**				
Male	3,070	212	1 [Reference]	
Female	3,787	356	1.41 (1.15–1.74)	.001
**Race/ethnicity**				
Non-Hispanic white	5,947	506	1 [Reference]	
Hispanic	575	44	0.93 (0.64–1.34)	.68
Non-Hispanic black	57	4	0.57 (0.19–1.77)	.34
Asian	145	10	1.22 (0.61–2.46)	.57
Other	138	9	0.74 (0.35–1.58)	.44
**Insurance**				
Commercial	3,967	294	1 [Reference]	
Medicare	2,208	194	0.86 (0.65–1.14)	.29
Medicaid	272	21	0.77 (0.45–1.29)	.32
Uninsured	415	64	1.47 (0.97–2.24)	.07
**Clinical Characteristics**				
**Prediabetes duration, y**				
<5	5,389	458	1 [Reference]	
5–9	1,303	100	0.71 (0.55–0.92)	.01
≥10	170	15	0.96 (0.53–1.75)	.90
**Chronic conditions^b^**				
Depression	2,808	272	1.13 (0.93–1.39)	.23
Coronary artery disease	1,234	114	1.02 (0.79–1.33)	.86
Congestive heart failure	516	51	0.93 (0.63–1.36)	.70
Atrial fibrillation	416	45	1.30 (0.89–1.89)	.18
High blood pressure	3,908	336	1.15 (0.89–1.47)	.28
**Medication class^c^**				
Antihypertensive	3,322	274	0.77 (0.60–0.99)	.04
Atypical neuroleptic	576	55	0.99 (0.71–1.41)	.99
Metformin	1,110	124	1.32 (1.04–1.68)	.02
Statin	3,497	304	1.03 (0.84–1.26)	.79
**Weight at study enrollment, kg**	6,862	573	1.01 (1.00–1.01)	.03
**BMI class at study enrollment (kg/m^2^)**				
Underweight (<18.5)	40	2	0.61 (0.13–2.92)	.54
Normal (18.5–24.9)	686	38	1 [Reference]	
Overweight (25.0–29.9)	1,679	130	1.37 (0.94–1.99)	.09
Obese (≥30.0)	4,193	355	1.17 (0.79–1.75)	.43
**Clinic Characteristics**				
**No. of patients attributed to clinics**	6,862	573	1.00 (0.99–1.00)	.74
**No. of providers per clinic**	6,862	573	0.99 (0.94–1.04)	.60
**Urban location of clinic**				
No	1,276	118	1 [Reference]	
Yes	5,013	455	1.24 (0.56–2.78)	.60
**Level of medical home implementation^d^**				
None	1,083	147	1 [Reference]	
Planning	449	27	0.57 (0.20–1.63)	.29
Adoption	1,733	115	0.48 (0.23–1.01)	.05
Routinized	3,024	284	0.59 (0.36–0.98)	.04

Abbreviations: CI, confidence interval; DPP, diabetes prevention program; OR, odds ratio.

a After all inclusion and exclusion criteria were applied to 17,142 patients who had a diagnosis of prediabetes (HbA1c of 5.70%–6.49% or fasting plasma glucose of 100–125 mg/dL) recorded in the Intermountain Healthcare enterprise data warehouse. Numbers in each category may not add to 6,862 because data were missing for some items.

b The reference group for each chronic condition is the group of patients who did not have the condition.

c The reference group for each medication class is the group of patients who were not taking the medication.

d Clinics were annually classified by leadership using a scorecard administered according to the standardized mental health integration care process model and a modified patient-centered medical home assessment based on National Committee for Quality Assurance recognition (26).

**Effectiveness.** DPP participants were 70% more likely to achieve a 5% weight loss than were nonparticipants (OR, 1.70; 95% confidence interval [CI], 1.29–2.25; *P* < .001) after data were adjusted for possible confounders. DPP participants were also less likely to have an incident diagnosis of type 2 diabetes during the study period (OR, 0.49; 95% CI, 0.28–0.86; *P* = .01). DPP participants lost more weight (but not significantly more) than nonparticipants (β = −1.36; 95% CI, −2.76 to 0.05; *P* = .058).

**Adoption.** The DPP was implemented in 7 of 8 regions (Table 3). The extensive geography of the nonparticipating region would have made in-person counseling challenging, so this region declined to participate. Of 63 clinics, 174 providers at 53 clinics referred patients to the DPP. The number of referrals per provider ranged from 1 to 32, and the number DPP participants varied by region from 31 to 163.

**Table 3:** Summary Measures of Adoption and Implementation of a Diabetes Prevention Program (DPP), by Region^a^, Intermountain Healthcare, 2013–2014

Measures	By Region^a^							Total
	1	2	3	4	5	6	7	
**Adoption**								
Eligible^b^ prediabetes patients, n	603	1,236	1,205	944	791	597	1,486	6,862
DPP participants, n (%)	34 (5.6)	163 (13.2)	48 (4.0)	31 (3.3)	84 (10.6)	75 (12.6)	138 (9.3)	573 (8.4)
DPP-referring clinics of clinics in region, n (%)	4 of 5 (80)	8 of 9 (89)	5 of 9 (56)	9 of 9 (100)	8 of 9 (89)	10 of 13 (77)	9 of 9 (100)	53 of 63 (84)
DPP-referring providers, n	15	44	21	19	22	22	31	174
Range of patients referred per provider	1–5	1–32	1–5	1–4	1–12	1–14	1–16	1–32
**Implementation**								
DPP counseling encounters^d^ per participant, mean (range), n	2.1 (1–12)	2.7 (1–15)	3.3 (1–15)	2.3 (1–12)	2.5 (1–15)	1.5 (1–14)	1.9 (1–16)	2.3 (1–16)
DPP participants with only 1 encounter, n (%)	26 of 34 (77)	91 of 163 (56)	28 of 48 (58)	18 of 31 (58)	44 of 84 (52)	54 of 75 (72)	117 of 138 (85)	378 of 573 (66)
W2H^c^ participants with ≥12 encounters, n (%)	3 of 4 (75)	13 of 20 (65)	7 of 9 (78)	2 of 3 (67)	5 of 7 (71)	1 of 4 (25)	4 of 7 (57)	35 of 54 (65)

a Intermountain Healthcare is organized into 8 regions, based on travel and referral patterns. One region declined to participate.

b After all inclusion and exclusion criteria were applied to 17,142 patients who had a diagnosis of prediabetes (HbA1c of 5.70%–6.49% or fasting plasma glucose of 100–125 mg/dL) recorded in the Intermountain Healthcare enterprise data warehouse.

c W2H, Weigh to Health, a hospital-based behavioral program, offered in 12 classes during 6 months.

d Encounters were defined as the number of visits during the DPP program.

**Implementation.** The mean number of DPP counseling encounters per patient was 2.3 (range, 1–16) and varied by region (Table 3). Sixty-six percent of participants had only 1 DPP counseling encounter (range for regions, 52%–85%); of 54 W2H participants, 35 (65%) had 12 or more encounters (range for regions, 25%–78%).

## Discussion

Overall, the first-year results from the Intermountain Healthcare DPP demonstrated encouraging potential for translating DPP-based interventions into primary care clinics. Although only 8.4% of patients with prediabetes participated in the DPP, we found a significant association with achieving a 5% weight loss and reduction in type 2 diabetes incidence among participants compared with nonparticipants. We found that female sex, age of 70 years or older, overweight, depression, higher weight at study enrollment, and a prescription for metformin were associated with a greater likelihood of participation. The program was broadly adopted by clinics and providers in the Intermountain Healthcare system. Although the mean number of DPP participants per provider was low (48% referred <5 patients), several providers were champions of the program. Few patients participated in more than 1 intervention option, and most had only 1 encounter during the study period.

Our results support research from other DPP-based interventions deployed in similar delivery systems such as Kaiser Permanente Colorado (20) and the Department of Veterans Affairs (30). Preliminary results from a national model of diabetes prevention linking health insurers and community programs suggest that “large-scale prevention efforts can be effective, scalable and sustainable with collaboration, health information technology, community-based delivery of evidence-based interventions, and novel payment structures” (31). While previous studies laid the foundation for translating diabetes prevention into care delivery, our study demonstrated support from organizational leadership and providers in enrolling patients in the program and revealed promising effectiveness of an intervention with multiple treatment options.

One potentially unique finding from our study suggested that the program was broadly adopted by providers and leadership across the system. However, referral rates and program fidelity varied across regions. Although our DPP was built on long-standing quality-improvement theory, lack of electronic medical record alerts, staff turnover, continuous provider training, and clinic resources may have contributed to differences in outcomes by region. By leveraging factors associated with successful program implementation, such as provider leadership and education, defined workflow process, system strategy and prioritization, health information technology, and payment mechanisms that support program access, the program steadily increased the number of participants during the study period. Continuous quality improvement was used to develop and refine the program. A dedicated and interdisciplinary team of clinicians, administrators, and researchers was involved from the program’s inception and drove the plan–do–study–act cycles that improved it. This approach included regular reporting back to clinical leaders and clinicians on patient referral and participation, further reinforcing their critical role in promoting and championing diabetes prevention.

Future plans call for expanded measurement, including developing a prediabetes registry, defining and evaluating patient engagement, and tailoring interventions for priority populations to achieve DPP goals. Production of a systems-based strategic plan, which includes coordination with community-based and employer-based programs and insurance companies while aligning financial incentives for patients and clinical providers, will continue to redefine the program. In addition, understanding the role of technology in delivering online lifestyle counseling may offer additional solutions for diabetes prevention.

This study has several limitations. Patients were not randomly assigned to participate in the intervention and, therefore, motivation to participate or greater readiness to change behavior may explain associations in participation or the weight-loss differences observed. This evaluation was performed during a short period after enrollment; further longitudinal study is needed to determine the sustainability of the program’s impact over a prolonged period. Although the methods used in this study attempted to account for differences in reach and adoption of the program across the Intermountain Healthcare clinics where the patients received care, they may not have accounted for all differences in practice that could have affected the results.

Study groups were selected according to established definitions. However, data could have been miscoded, thereby creating selection bias. Patients with a diagnosis of type 2 diabetes were excluded from the study, yet a diagnosis could have been overlooked and the patient not excluded because of the definitions used or because care for the condition could have occurred outside of the Intermountain Healthcare system.

Our findings may not be generalizable to populations outside of Intermountain Healthcare because of differences in patient characteristics, local implementation, and resources allocated. Information was not available on weight-loss activities outside of the Intermountain Healthcare DPP, which potentially could differ between participants and nonparticipants. Finally, social determinants of health, such as where the patient was born, their living conditions, and education and income levels have also been associated with health outcomes, but these data were not available for study.

DPP-based interventions deployed in Intermountain Healthcare’s delivery system demonstrated moderate effectiveness in the short term, but the participation rate among eligible patients was low. Broad adoption across regions by providers and leadership demonstrated organizational buy-in, and much of the clinical effect resulted from interventions that were less program-intensive than interventions described in landmark studies of the National Diabetes Prevention Program.

## Appendix Definitions of Chronic Conditions Used in Study of Diabetes Prevention Program, Intermountain Healthcare, 2013–2014

**Table:**

Chronic Condition	Diagnoses (ICD-9-CM^a^)	Encounters (CPT^a^)	Exclusions
High blood pressure	360.42, 362.11, 401, 401.0, 401.1, 401.9, 402, 402.0, 402.00, 402.01, 402.1, 402.10, 402.11, 402.9, 402.90, 402.91, 403, 403.0, 403.00, 403.1, 403.10, 403.9, 403.90, 404, 404.0, 404.00, 404.01, 404.1, 404.10, 404.11, 404.90, 404.9, 404.91, 405, 405.0, 405.01, 405.09, 405.1, 405.11, 405.19, 405.9, 405.91, 405.99, 437.2	Outpatient visit with 99201–05, 99211–15, 99241–45, 99341–50, 99381–87, 99391–97, 99401–04, 99411–12, 99420, 99429, or 99455–56	No documentation of renal transplant
Atrial fibrillation	427.31	Inpatient admission with either 3734 or 3726–28	None
Coronary artery disease	410.xx, 411.0, 411.1, 411.81, 411.89, 412.0, 413.0, 413.9, 414.0, 414.01, 414.02, 414.03, 414.04, 414.05, 414.06, 414.07, 414.11, 414.80, 414.90	None	None
Congestive heart failure	398.91, 402.01, 402.11, 402.91, 404.01, 404.03, 404.11, 404.13, 404.91, 404.93, 428.0, 428.1, 428.20, 428.21, 428.22, 428.23, 428.30, 428.31, 428.32, 428.33, 428.40, 428.41, 428.42, 428.43, 428.9	None	None
Depression	296.2, 296.20, 296.21, 296.22, 296.23, 296.24, 296.25, 296.26, 296.3, 296.30, 296.31, 296.32, 296.33, 296.34, 296.35, 296.36, 296.82, 296.90, 298, 298.0, 300.4, 309.1, 309.28, 311	Hospital admission *or* emergency department visit *or* outpatient visit with 99201–05, 99211–15, 99241–45, 99341–50, 99381–87, 99391–97, 99401–04, 99411–12, 99420, 99429, or 99455–56	None

Abbreviations: CPT, current procedural terminology; ICD-9-CM, *International Classification of Diseases, Ninth Revision, Clinical Modification*.

^a^ To be identified with a chronic condition, specifications require at least 1 CPT and ICD-9-CM code to be paired on the same day.
